# Visualizing atomic structure and magnetism of 2D magnetic insulators via tunneling through graphene

**DOI:** 10.1038/s41467-020-20376-w

**Published:** 2021-01-04

**Authors:** Zhizhan Qiu, Matthew Holwill, Thomas Olsen, Pin Lyu, Jing Li, Hanyan Fang, Huimin Yang, Mikhail Kashchenko, Kostya S. Novoselov, Jiong Lu

**Affiliations:** 1 Department of Chemistry, National University of Singapore, 3 Science Drive 3, Singapore, 117543 Singapore; 2 National Graphene Institute, University of Manchester, Manchester, M13 9PL UK; 3 Computational Atomic-scale Materials Design (CAMD), Department of Physics, Technical University of Denmark, 2800 Kgs, Lyngby, Denmark; 4 Centre for Advanced 2D Materials (CA2DM), National University of Singapore, 6 Science Drive 2, Singapore, 117546 Singapore; 5 Center for Photonics and 2D Materials, Moscow Institute of Physics and Technology, Dolgoprudny, 141700 Russia; 6 Department of Materials Science & Engineering, National University of Singapore, 9 Engineering Drive 1, Singapore, 117575 Singapore; 7 Chongqing 2D Materials Institute, Liangjiang New Area, Chongqing, 400714 China

**Keywords:** Information storage, Electronic properties and devices

## Abstract

The discovery of two-dimensional (2D) magnetism combined with van der Waals (vdW) heterostructure engineering offers unprecedented opportunities for creating artificial magnetic structures with non-trivial magnetic textures. Further progress hinges on deep understanding of electronic and magnetic properties of 2D magnets at the atomic scale. Although local electronic properties can be probed by scanning tunneling microscopy/spectroscopy (STM/STS), its application to investigate 2D magnetic insulators remains elusive due to absence of a conducting path and their extreme air sensitivity. Here we demonstrate that few-layer CrI_3_ (FL-CrI_3_) covered by graphene can be characterized electronically and magnetically via STM by exploiting the transparency of graphene to tunneling electrons. STS reveals electronic structures of FL-CrI_3_ including flat bands responsible for its magnetic state. AFM-to-FM transition of FL-CrI_3_ can be visualized through the magnetic field dependent moiré contrast in the d*I*/d*V* maps due to a change of the electronic hybridization between graphene and spin-polarised CrI_3_ bands with different interlayer magnetic coupling. Our findings provide a general route to probe atomic-scale electronic and magnetic properties of 2D magnetic insulators for future spintronics and quantum technology applications.

## Abstract (web summary)

In this work Qiu et al. demonstrate that the application of van der Waals technology to STM/STS will dramatically expand the capabilities of the latter, allowing STM/STS to investigate the structure, electronic properties and magnetism of 2D magnetic insulators at the atomic scale.

## Introduction

Scanning tunneling microscopy (STM) is a versatile tool when it comes to the study of electronic properties of metals at the atomic scale. Despite its obvious advantages, this technique also has a number of drawbacks: it can only investigate conductive materials and lacks direct access to the information about the momentum distribution and magnetic ordering of the electronic states. Recently, engineering vdW heterostructures promoted itself as a versatile tool to modify and study the electronic and magnetic structures of various 2D materials: insulators, semiconductors, metals, superconductors, and ferromagnets^1–5^. Here, we demonstrate that the application of vdW technology to the STM will dramatically expand the capabilities of the latter, allowing it to study insulating materials and gaining information about the magnetic ordering in 2D ferromagnets^6–9^.

To this end, we assemble vdW heterostructures based on investigated 2D materials covered with monolayer graphene. Graphene, being conductive, ideally suits STM. At the same time, its low density of states (DOS) and the ability of its electronic states to hybridize with the electronic states from other 2D materials allow for gaining information about materials buried underneath at the atomic scale^10–13^. Furthermore, the projection of the electronic states of other materials on graphene depends strongly on the atomic arrangements; thus, additional information (like stacking between buried layers, or even information about magnetic structure) can be extracted from the close examination of the moiré structure between graphene and materials under study.

## Results

### Structural characterization of graphene/CrI_3_/graphite

**Fig. 1:**
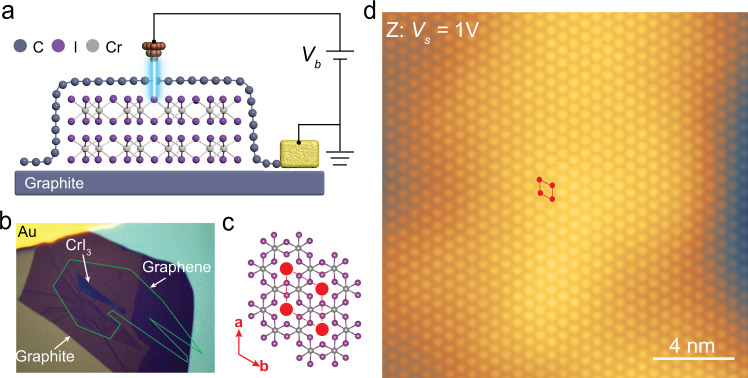
The vdW heterostructure of G/FL-CrI_3_/Gr for STM study. **a** The schematic illustration and **b** the optical image of our experimental setup. Our sample consists of monolayer graphene covering FL-CrI_3_ stacking on graphite flake (G/FL-CrI_3_/Gr). **c** The atomic structure of monolayer CrI_3_ (top view). **d** Large-size STM topographic image of G/FL-CrI_3_/Gr (*V*_*s*_ = 1 V, *I*_*t*_ = 0.1 nA).

Here, we used STM to study mechanically exfoliated FL-CrI_3_ sandwiched between a top graphene layer and a bottom graphite thin flake (G/FL-CrI_3_/Gr). The schematic illustration of our experimental setup with the corresponding optical image of G/FL-CrI_3_/Gr heterostructure is presented in Fig. 1a, b, respectively. We investigated CrI_3_ thin flakes with different thickness: from monolayer to few nm in thickness. A representative large-size STM topographic image of G/FL-CrI_3_/Gr (Fig. 1d) reveals a triangular lattice with a periodicity of 0.69 ± 0.01 nm, consistent with the reported lattice constant of CrI_3_^14^. Therefore, it is very likely that the underlying FL-CrI_3_ dominates the STM contrast at this particular sample bias *V*_*s*_ = 1V, which will be explained in detail later. The intact triangular lattice observed here indicates structural integrity of the underlying FL-CrI_3_ flake due to effective protection from the top graphene layer.

### Probing the electronic properties of G/CrI_3_/Gr

**Fig. 2:**
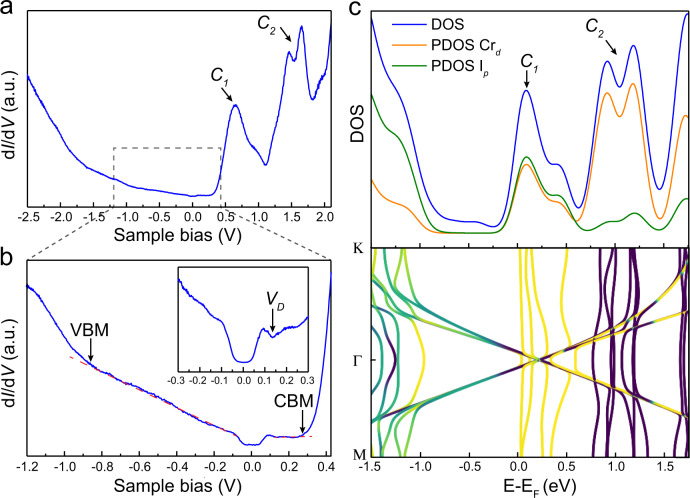
The electronic structure of G/CrI_3_. **a** The d*I*/d*V* spectrum of G/FL-CrI_3_/Gr taken in a large sample bias window (−2.5 V ≤ *V*_*s*_ ≤ 2.1 V). Two prominent double-peak features are indicated by *C*_1_ and *C*_2_, respectively. **b** The d*I*/d*V* spectrum of G/FL-CrI_3_/Gr taken in a small sample bias window (−1.2 V ≤ *V*_*s*_ ≤ 0.42 V). The band edges are indicated by VBM and CBM. The inset shows the d*I*/d*V* spectrum near the Fermi level (−0.3 V ≤ *V*_*s*_ ≤ 0.3 V). The local conductance minimum is indicated by *V*_*D*_. **c** Calculated density of states (DOS) and band structure of G/ML-CrI_3_ using Hubbard *U* = 0.5 eV. Both DOS and the projected DOS (PDOS) on iodine *p* orbitals and chromium *d* orbitals are shown. The color-coding in the band structure indicates the expectation value of spin *S*_*z*_ with yellow and purple corresponding to spin-up and -down, respectively.

We then explored local electronic structures of G/FL-CrI_3_/Gr using scanning tunneling spectroscopy (STS). d*I*/d*V* spectrum taken over G/FL-CrI_3_/Gr (Fig. 2a) reveals two prominent double-peak features above Fermi level (*E*_*F*_), which are labeled as *C*_1_ (0.3 V < *V*_*s*_ < 1.1 V) and *C*_2_ (1.1 V < *V*_*s*_ < 1.8 V), respectively. A close examination of the d*I*/d*V* spectrum in combination with bias-dependent and tunneling current-dependent STM images (Fig. S1 and Fig. S2) allows us to identify the band edges as well as the bandgap of FL-CrI_3_. We tentatively assign the kink around *V*_*s*_ = −0.87 V to the valence band maximum (VBM) of CrI_3_ and the steep rise at *V*_*s*_ = 0.26 V to the conduction band minimum (CBM), which yields a bandgap of 1.13 eV for FL-CrI_3_, consistent with the reported values obtained by optical measurements^15^. Within the bandgap of FL-CrI_3_, the d*I*/d*V* signal is mainly contributed by graphene as manifested by three characteristic features: (i) a nearly linear DOS as reflected by d*I*/d*V* in the sample bias range of −0.8 V < *V*_*s*_ < −0.2 V, (ii) a gap-like feature around *E*_*F*_ with a sharp increase in d*I*/d*V* around |*V*_*s*_| = 63 mV owing to the suppression of the tunneling current due to momentum mismatch and phonon-assisted inelastic tunneling^16^, and (iii) a local conduction minimum around *V*_*S*_ = 0.13 V associated with the Dirac point of graphene (*E*_*D*_)^16^. For monolayer CrI_3_ (ML-CrI_3_), the d*I*/d*V* spectrum of G/ML-CrI_3_/Gr closely resembles that of G/FL-CrI_3_/Gr (Fig. S3), presumably due to a weak layer dependence of CrI_3_ electronic structures^17^.

### Band structure calculations

To gain better insight into the electronic structures of G/CrI_3_, we have performed spin-polarized band structure calculations using the Hubbard-corrected local density approximation (LDA + U). The calculations employed a (5 × 5) supercell of graphene placed on a ($\sqrt 3 \times \sqrt 3$) supercell of ML-CrI_3_. The calculated band structure (Fig. 2c) shows that the flat bands of CrI_3_ hybridize with graphene Dirac cones in the majority-spin channel and electrons transfer from graphene to CrI_3_, in good agreement with previous density–functional theory (DFT) calculations of G/CrI_3_^18–20^. A direct comparison of the experimental d*I*/d*V* spectra with the theoretical DOS reveals that two double-peak features (*C*_1_ and *C*_2_) in the d*I*/d*V* spectra arise from relatively flat conduction bands of G/CrI_3_. Specifically, *C*_1_ is contributed by majority-spin states (0 eV < *E* − *E*_*F*_ < 0.5 eV) with a nearly equal contribution from Cr *d* states and I *p* states, both of which strongly hybridize with majority-spin Dirac cones. In contrast, *C*_2_ is contributed mainly by minority-spin *d* states of Cr (0.8 eV < *E* − *E*_*F*_ < 1.3 eV) with a negligible hybridization with graphene states.

We note that the Hubbard U has a negligible influence on the overall band shape but significantly changes the energy spacing between *C*_1_ and *C*_2_. It is found that the use of a Hubbard U of 0.5 eV yields an energy spacing around 0.8 eV between *C*_1_ and *C*_2_, in good agreement with that observed in the d*I*/d*V* spectra (Fig. S4). The bandgap of ML-CrI_3_ predicted from spin-polarized DFT calculations is around 1.24 eV, close to the bandgap measured experimentally. Based on calculated band structures of G/CrI_3_ (Fig. 2c), it is noted that *E*_*D*_ lies in the bottom of conduction bands of CrI_3_ in contrast to our experimental observation that *E*_*D*_ is located inside the bandgap. Such a discrepancy is attributed to the additional charge transfer between the bottom graphite substrate and G/CrI_3_.

**Fig. 3:**
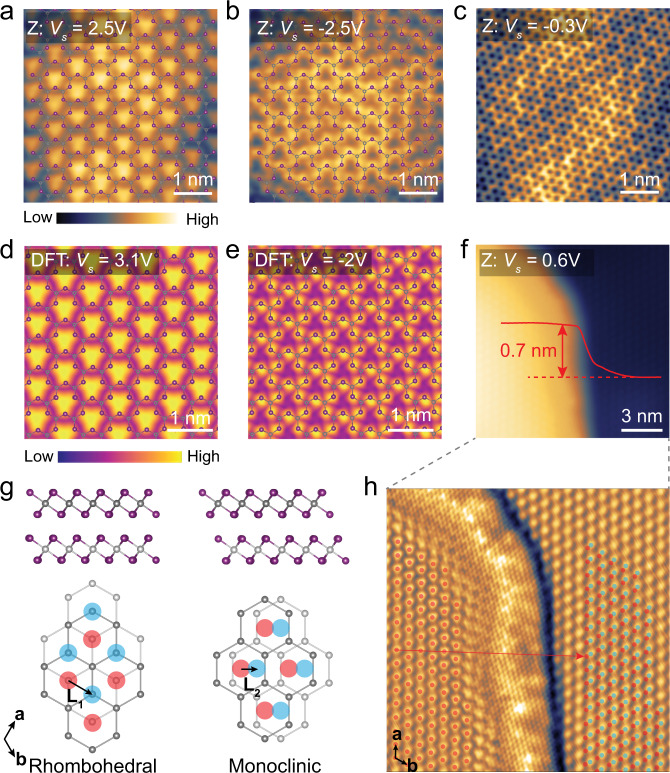
STM measurements of G/FL-CrI_3_/Gr. **a**–**c** Bias-dependent STM images of G/FL-CrI_3_/Gr. STM images taken at (**a**) *V*_*s*_ = 2.5 V and (**b**) at *V*_*s*_ = −2.5 V with the superimposed atomic structure of ML-CrI_3_ (I atoms on the bottom atomic plane are removed for clarity). STM images taken at (**c**) *V*_*s*_ = −0.3 V. The tunneling current is *I*_*t*_ = 1 nA. **d**, **e** Simulated STM images taken at (**d**) *V*_*s*_ = 3.1 V and (**e**) *V*_*s*_ = −2 V with the superimposed atomic structure of ML-CrI_3_. **f** STM image acquired across the single-layer step of CrI_3_ (*V*_*s*_ = 0.6 V, *I*_*t*_ = 0.2 nA). **g** The atomic structure of adjacent CrI_3_ layers with rhombohedral stacking and monoclinic stacking. The upper (lower) panels are side (top) views. The top view shows the honeycomb lattice formed by Cr atoms (I atoms are removed for clarity), where the center of each hexagon in the upper (lower) layer is indicated by the red (blue) circle. **h** The processed STM image of **f** by using edge enhancement filters to better visualize the atomic lattice of both layers. The lattice of the upper (lower) layer is represented by the red (blue) circle. To intuitively show the atomic translation between two layers, a replica of the upper-layer lattice (translated by (8*a* + 16*b*) with respect to the original lattice of the upper layer) is shown as the red circle on the lower layer. The red arrow represents the vector (8*a* + 16*b*).

We also found that graphene and the underlying CrI_3_ lattice can be selectively imaged by choosing an appropriate sample bias. Figure 3a–c shows three representative bias-dependent STM images taken on G/FL-CrI_3_/Gr (a full set of bias-dependent STM images is shown in Fig. S1). The honeycomb lattice of graphene can be clearly resolved at low sample bias (*V*_*s*_ = −0.3 V) within the bandgap (Fig. 3c), while CrI_3_ lattice with two distinct patterns can be imaged at large sample biases outside the bandgap (Fig. 3a, b). STM image acquired at *V*_*s*_ = 2.5 V (Fig. 3a) shows a periodic triangular “cluster” pattern with a lattice constant of 0.69 ± 0.01 nm. We then superimposed the atomic structure of ML-CrI_3_ over the corresponding STM image in Fig. 3a (note that the bottom I atoms in the atomic model are removed for clarity) for a close examination, which reveals that individual triangular clusters are formed by three nearest I atoms in the top atomic plane. In addition, the maxima of triangular cluster protrusion are located at the center of three nearest I atoms in the top atomic plane, equivalent to the center of the hexagon formed by six adjacent Cr atoms. This is similar to the reported STM image of CrBr_3_^21^. By contrast, the STM image taken at *V*_*s*_ = −2.5 V (Fig. 3b) shows that the maxima of the protrusion are nearly located over the I atoms in the top atomic plane.

Both STS and STM results indicate that graphene is almost transparent to tunneling electrons when the sample bias is outside the bandgap of FL-CrI_3_. Otherwise, it would not be possible to probe the atomic structures and electronic properties of the underlying insulating CrI_3_ flake as semimetallic graphene is closer to the tip by ~3.5 Å (predicted by DFT calculations). Such transparency of graphene in the tunneling process has been observed for graphene grown on metallic substrate^11–13^. It turns out that the substrate states can extend further beyond graphene because graphene’s *π* states are strongly localized by both the large in-plane wave vector of graphene’s *π* states and the small out-of-plane extension of their atomic orbitals^11^. In the case of G/CrI_3_ heterostructure, our DFT calculations also confirm that the CrI_3_ states dominate the simulated STM images at a distance around 4 Å above graphene surface (refer to supporting information S4 for more details). However, the spatial structure of wavefunctions in the regions of space where they have decayed by more than a factor of 10^6^ becomes unreliable (Fig. S5). Therefore, we have chosen to focus on STM simulations at distances of 3 Å above the graphene layer with the introduction of a damping weightage to mimic the structure at larger distances. The simulated STM images at both positive and negative sample biases (Fig. 3d, e) show good agreement with the corresponding experimental STM images (Fig. 3a, b).

### Visualizing the stacking order in the exfoliated FL-CrI_3_

Stacking-dependent interlayer magnetism is another peculiar feature in 2D magnetic insulators. It has been predicted that the monoclinic stacking favors interlayer antiferromagnetic (AFM) coupling, while the rhombohedral stacking favors the interlayer ferromagnetic (FM) coupling^15,17,22–24^. Bulk CrI_3_ undergoes a structural phase transition from a monoclinic to a rhombohedral phase at 220 K accompanied with the interlayer FM coupling below the critical temperature of 61 K^14^. By contrast, various reports have suggested that exfoliated FL-CrI_3_ thin flakes show interlayer AFM coupling below the critical temperature^2,6,20,25–29^. This was interpreted as FL-CrI_3_ exfoliated at room temperature is kinetically trapped in the monoclinic phase upon cooling (which favors interlayer AFM coupling)^17,22,29^. Such a hypothesis is further verified in recent works by monitoring the change in the second harmonic generation of bilayer CrI_3_ during its AFM-to-FM transition^30^ and phase-sensitive Raman modes of FL-CrI_3_^29^. However, direct atomic-scale visualization of the low-temperature phase of exfoliated FL-CrI_3_ is still lacking.

Here, we managed to directly visualize the monoclinic stacking in exfoliated FL-CrI_3_ at low temperature by imaging the lateral translation between adjacent CrI_3_ layers. Figure 3g illustrates the top and side views of adjacent CrI_3_ layers with the rhombohedral and monoclinic stacking. As shown in Fig. 3g, the lower CrI_3_ layer is laterally translated by ${L}_1 = \frac{1}{3}{a} + \frac{2}{3}{b}$ (${L}_2 = \frac{1}{3}{a} + \frac{1}{3}{b}$) with respect to the upper CrI_3_ layer for the rhombohedral (monoclinic) stacking^17,29^. The top view shows the honeycomb lattice formed by Cr atoms (I atoms are removed for clarity) for two different stacking phases, where the center of each hexagon is indicated by the red (blue) circle in the upper (lower) CrI_3_ layer. As shown in Fig. 3a, the center of each hexagon formed by six adjacent Cr atoms appears as a protrusion in the STM image taken at the positive sample bias. This allows us to identify the lateral translation between adjacent CrI_3_ layers by examining the STM images of both upper and lower CrI_3_ layer across a single-layer step. Figure 3f presents a typical STM image of a single-layer step in CrI_3_ with an expected apparent step height of 0.7 ± 0.1 nm^6^. The CrI_3_ lattice in both upper and lower layers can be better visualized in the STM image processed by the edge enhancement filter in SPIP (Fig. 3h)^31^. We then identify the lattice of both upper and lower layers, which are represented by the red and the blue circles, respectively (Fig. 3h). A statistical analysis shows that the lattice of the lower layer is translated by *L* = (8.35 ± 0.08)*a* + (16.36 ± 0.06)*b* with respect to the lattice of the upper layer. Taking the modulus of the translation vector *L*, the lower layer is determined to be translated by (0.35 ± 0.08)*a* + (0.36 ± 0.06)*b* with respect to the upper layer, which reveals the monoclinic stacking in exfoliated FL-CrI_3_ at low temperature within the experimental uncertainty. Such a stacking favors the interlayer AFM coupling as predicted by theory^17,22–24^.

### Probing the magnetic properties of G/FL-CrI_3_/Gr

**Fig. 4:**
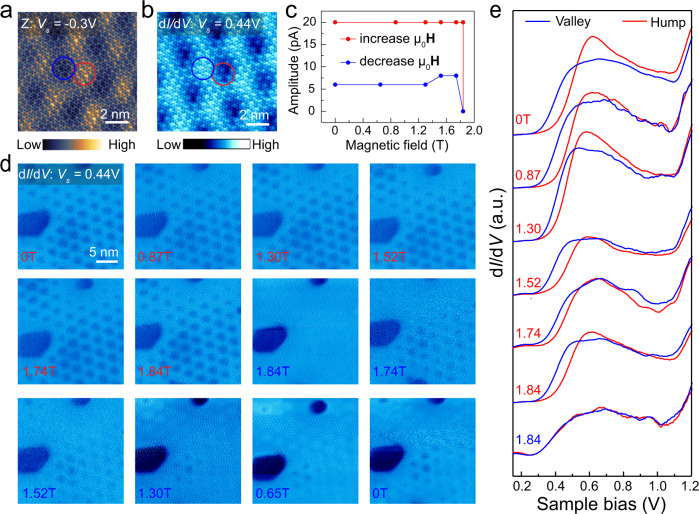
Magnetic field-dependent moiré contrast in *dI*/*dV* maps. **a** STM image (*V*_*s*_ = −0.3 V, *I*_*t*_ = 0.2 nA) shows the moiré pattern of G/FL-CrI_3_/Gr. The lower (higher) region is referred as moiré valley (hump) indicated by the blue (red) circle. **b** The corresponding d*I*/d*V* map (*V*_*s*_ = 0.44 V, *I*_*t*_ = 0.5 nA). **c** Magnetic field-dependent moiré contrast in the d*I*/d*V* maps (*V*_*s*_ = 0.44 V, *I*_*t*_ = 0.5 nA). **d** Magnetic field-dependent d*I*/d*V* maps (*V*_*s*_ = 0.44V, *I*_*t*_ = 0.5 nA). **e** Magnetic field-dependent d*I*/d*V* spectra taken at moiré valley (blue) and hump (red).

Apart from the structural and electronic properties of CrI_3_, we also found that the magnetic properties of underlying FL-CrI_3_ can be probed through graphene using magnetic field-dependent STM/STS measurements. STM image of G/FL-CrI_3_/Gr acquired at *V*_*s*_ = −0.3 V (Fig. 4a) exhibits the moiré superlattice with a periodicity of 3.14 ± 0.01 nm (refer to the supporting information S5 for more details). The dark (lower) and bright (higher) regions in the topographic STM image are defined as moiré valley and moiré hump, respectively (Fig. 4a). At zero magnetic field, the d*I*/d*V* spectra taken in valley and hump regions show a noticeable difference in terms of the energy position and peak intensity around *C*_*1*_ states (Fig. 4e). It is noted that *C*_1_ states result from the hybridization of majority-spin CrI_3_ and graphene states. Therefore, it is very likely that the spatial variation of *C*_1_ states in valley and hump regions is originated from the atomic registry-dependent hybridization between graphene and the underlying CrI_3_^32^. The d*I*/d*V* map taken at *V*_*s*_ = 0.44 V also captured a spatial moiré modulation of the LDOS (Fig. 4b), consistent with the d*I*/d*V* spectroscopic measurement. We then swept the vertical magnetic field and monitored the moiré contrast in the d*I*/d*V* maps. As shown in Fig. 4c, d, the characteristic moiré contrast with a nearly constant relative amplitude (defined as the difference in the d*I*/d*V* signal between moiré valley and hump as shown in Fig. S7) retains when the magnetic field gradually increases up to 1.84 T. We then ramped the sample bias from 2.2 V to −2.2 V to perform point d*I*/d*V* spectroscopy (Fig. S8a). During the measurement, we observed a sudden change of the *I*–*V* and d*I*/d*V* signal (Fig. S8b). By rescanning the same area at 1.84 T, we found that the characteristic moiré contrast vanished in the d*I*/d*V* map (*V*_*s*_ = 0.44 V) as shown in Fig. S8c. The magnetic field-dependent moiré contrast in d*I*/d*V* maps (taken at fixed bias *V*_*s*_ = 0.44 V) is consistent with the evolution of magnetic field-dependent full d*I*/d*V* spectra taken over moiré hump and moiré valley (Fig. 4e), which confirms its electronic origin. Upon exposing the sample to 1.84 T, the difference between the d*I*/d*V* spectra taken over moiré valley and hump vanishes and they become nearly identical, consistent with the disappearance of the moiré contrast in d*I*/d*V* maps. Interestingly, as the magnetic field gradually decreases, the moiré contrast reappears but with reduced relative amplitude, resulting in a forward and backward hysteresis (Fig. 4c, d). We note that the critical magnetic field to induce the change of moiré contrasts in different regions of the sample varies from 1.74 to 1.84 T (Figs. S9 and 10), presumably due to the variation of the local environment (like demagnetization field or the formation of domain structures)^6,28^.

## Discussion

The magnetic field-dependent moiré contrast in the d*I*/d*V* maps is likely to be associated with the AFM-to-FM transition in FL-CrI_3_: the critical magnetic field observed is very close to the typical magnetic field required to align all the spins in different layers of FL-CrI_3_ (Fig. S11)^2,15,20,29,33,34^. This hypothesis is further corroborated by our spin-polarized DFT calculation of the atomic registry-dependent band structure of G/four-layer CrI_3_ under AFM and FM interlayer coupling.

**Fig. 5:**
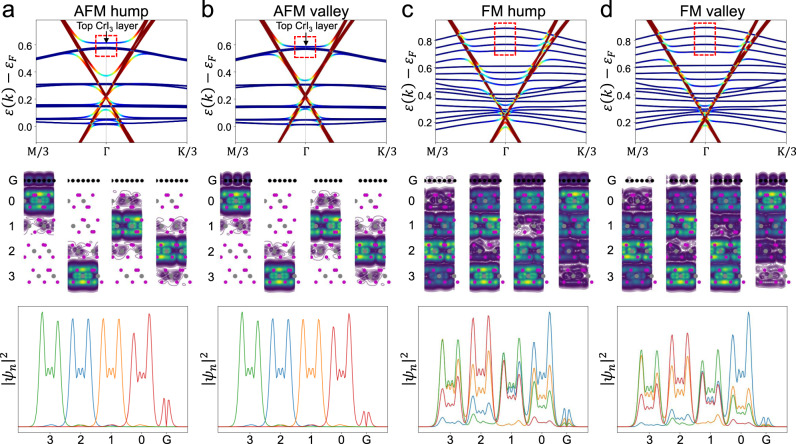
The atomic registry-dependent band structure of G/four-layer CrI_3_ under AFM and FM interlayer coupling. **a**–**d** The band structure and norm-squared wavefunctions of bands in G/four-layer CrI_3_ with FM and AFM interlayer order in the hump and valley geometries. The top panel shows the band structure of G/four-layer CrI_3_. We indicate four states using red dashed lines shown in each of the four cases. The middle panel shows contour plots of the norm-squared wavefunctions of indicated states averaged over the *y*-direction of the heterostructure. The bottom panel shows the norm-squared wavefunctions of indicated states averaged over the entire plane.

Figure 5 shows the calculated band structures of G/four-layer CrI_3_ (monoclinic phase) with two atomic arrangements (corresponding to the hump and valley) under two magnetic configurations (corresponding to FM and AFM interlayer coupling) (refer to S8 for more details). For the AFM interlayer coupling configurations, only the top CrI_3_ layer shows a noticeable electronic coupling to graphene in both moiré hump and valley regions, as visualized in Fig. 5a, b. The bands of individual CrI_3_ layers in AFM-coupled configuration are nearly degenerate and strongly localized in the individual CrI_3_ layer due to a weak interlayer hybridization (Fig. 5a, b). Because of this, only the bands of the top CrI_3_ layer hybridize with graphene states and are split off from bands of other CrI_3_ layers. A careful analysis of the band structures reveals larger band-splitting energy of the top CrI_3_ layer in moiré hump compared to moiré valley. This suggests that the electronic hybridization depends strongly on the local atomic registry between graphene and CrI_3_, which gives rise to a moiré contrast in d*I*/d*V* maps for G/CrI_3_ under AFM interlayer coupling.

In contrast, nearly all the four CrI_3_ layers are electronically coupled to graphene under the FM interlayer coupling configuration. This is because the bands from different CrI_3_ layers are delocalized over the entire structure (Fig. 5c, d). Although the hybridization still depends on the atomic registry between graphene and individual CrI_3_ layers, the moiré contrast created by each of the CrI_3_ layers in graphene is now shifted by a third of the moiré period (due to monoclinic stacking) and thus cancels each other. This explains why the transition to the FM state is seen as the disappearance of the moiré structure.

In conclusion, we have demonstrated a new approach to probe the atomic lattice, intrinsic electronic structure, and interlayer magnetism of mechanically exfoliated FL-CrI_3_ in a graphene-encapsulated vdW vertical heterostructure using STM/STS. Our results show that overlaid graphene not only protects exfoliated FL-CrI_3_ from degradation but also allows STM characterization of the underlying FL-CrI_3_ due to its peculiar transparency to tunneling electrons. The use of semimetallic graphene as a capping layer with electronic transparency to tunneling electrons fulfills the growing demand for the atomic-scale characterization of the artificially assembled vdW heterostructures based on air-sensitive 2D magnetic insulators toward next-generation spintronic devices.

## Methods

### Sample preparation

The sample is prepared using a well-established dry transfer technique in the glove box. The mechanically exfoliated FL-CrI_3_ flake is sandwiched between a top graphene layer and a bottom graphite flake. The thickness of FL-CrI_3_ flake is estimated to be ~8–10 nm via the analysis of the corresponding optical contrast. The sample is subjected to UHV annealing at 150 °C for 4 h to ensure the surface cleanness for the STM study.

### STM and STS measurements

Our STM and STS measurements were conducted at 4.5 K in the Createc LT-STM system with a base pressure lower than 10^–10^ mbar. The tungsten tip was calibrated spectroscopically against the surface state of Au(111) substrate. A superconducting coil was used to apply the out-of-plane external magnetic field with a maximum field of 1.84 T. All the d*I*/d*V* spectra were measured through a standard lock-in technique with a modulated voltage of 5–10 mV at the frequency of 700–900 Hz.

## Supplementary information

Supplementary Information

Peer Review File

## Data Availability

The data that support the findings of this study are available from the authors on reasonable request, see “Author contributions” for specific data sets.
