# Supplementary material for: Visualizing atomic structure and magnetism of 2D magnetic insulators via tunneling through graphene
Source: Nat Commun. 2021 Jan 4;12:70. doi: 10.1038/s41467-020-20376-w (PMC7782518; doi:10.1038/s41467-020-20376-w)
Supplement: Supplementary file 1 — Supplementary Information [file 41467_2020_20376_MOESM1_ESM.pdf]

Supplementary information

Table of Contents

Section S1. Sample bias dependent STM images of G/FL-CrI_3/Gr

Section S2. The STM images and STS spectra of G/ML-CrI_3/Gr

Section S3. Calculated DOS of G/ML-CrI_3 with different Hubbard U

Section S4. The transparency of graphene to tunneling electrons

Section S5. The moiré superlattice of G/FL-CrI_3/Gr at a twist angle $\varphi = 16°$

Section S6. The magnetic field dependent dI/dV maps of G/FL-CrI_3/Gr

Section S7. The reported magnetic field for AFM-FM transition in FL-CrI_3

Section S8. The atomic registry dependent electronic structures of G/four-layer CrI_3

Section S9. Methods for theoretical calculation

1. Sample bias dependent STM images of G/FL-CrI_3/Gr

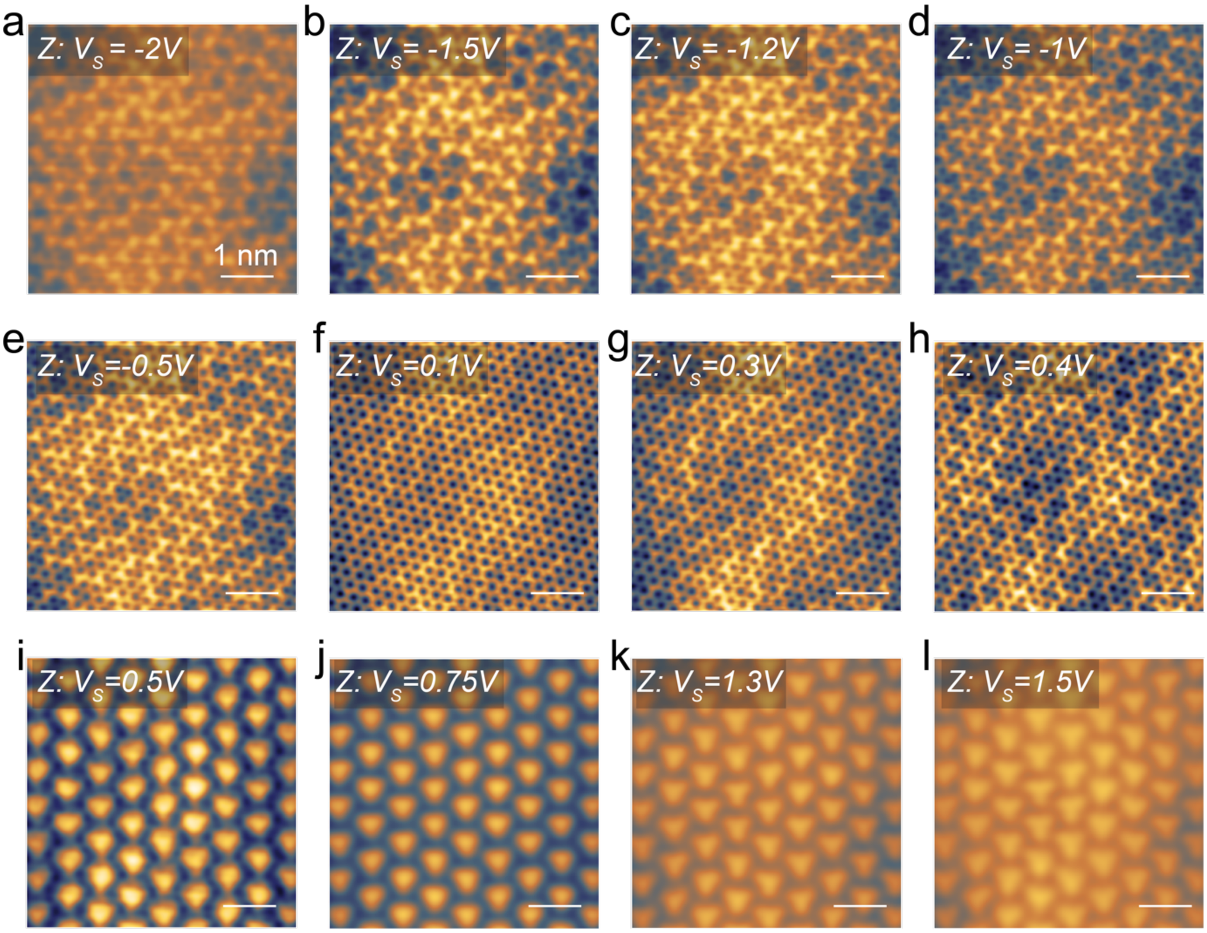

Figure S1. **Sample bias dependent STM images of the G/FL-CrI_3/Gr.** The tunneling current is 1 nA for all STM images.

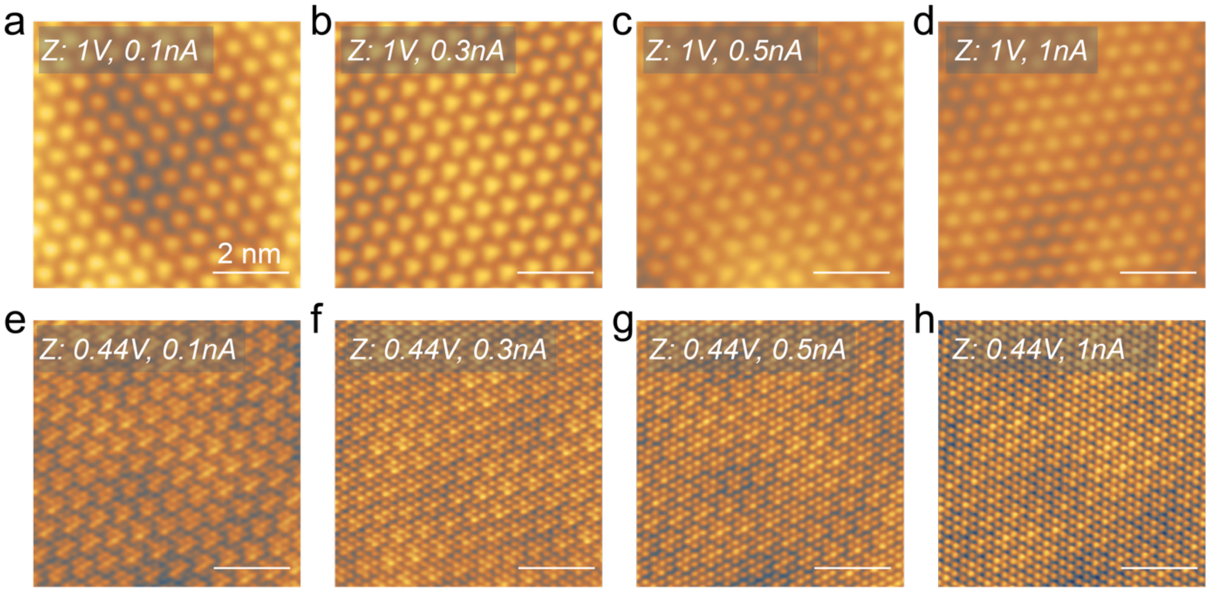

Figure S2. STM images of the G/FL-CrI_3/Gr recorded at varied tunneling current. (a-d) STM images ($V_s = 1$ V) recorded at varied tunneling current from 0.1 nA to 1 nA.Note the STM images could be taken on different areas. All these STM images resolves CrI_3 lattice, independent of tunnelling current. That is because the electronic states probed are deep into the CrI_3 conduction band and thus the CrI_3 states have a major contribution to the tunneling current. (e-h) STM images ($V_s = 0.44$V) recorded at varied tunneling current from 0.1nA to 1nA. A smaller positive bias ($V_s = 0.44$V) probes the electronic states near the CrI_3 conduction band edge as well as graphene states. In this case, the weight of integrated graphene states and integrated CrI_3 states may be comparable so that both graphene lattice and CrI_3 lattice can be resolved. A variation of tunneling current from 0.1 nA to 1 nA have a limited influence on the visualization of graphene lattice. An increase of tunneling current makes the graphene (CrI_3) lattice slightly more (less) prominent.

2. The STM images and STS spectra of G/ML-CrI_3/Gr

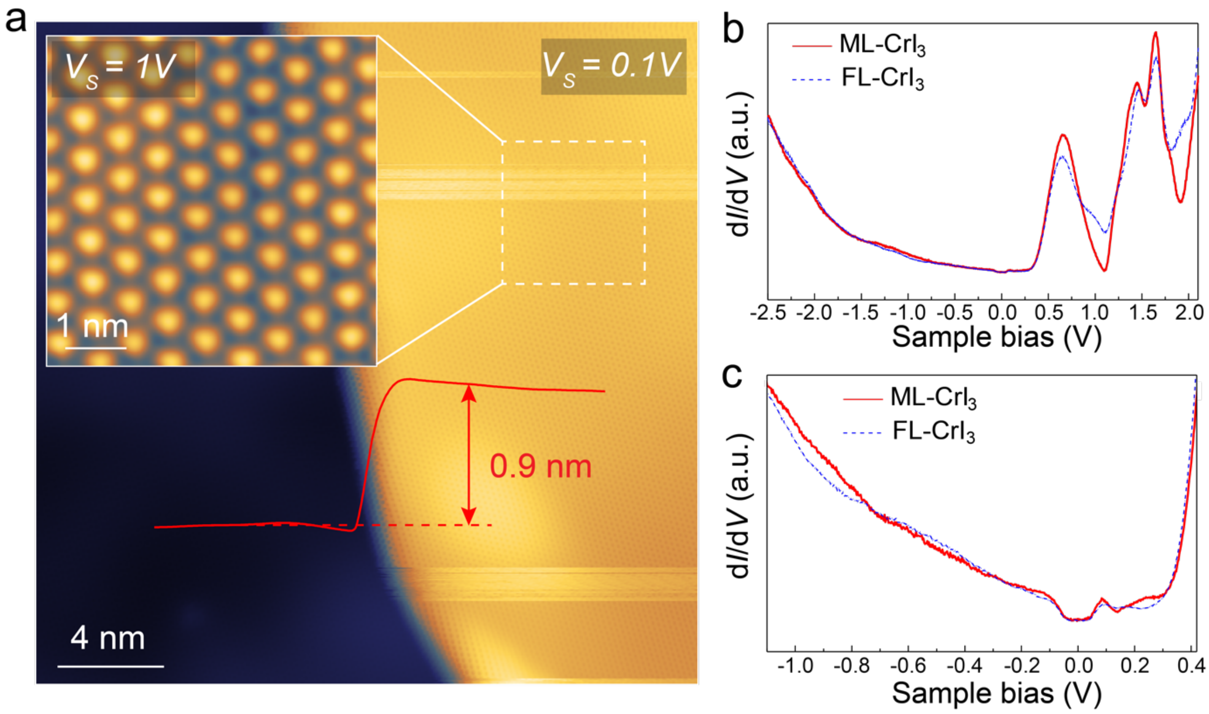

Figure S3. **The STM images and STS spectra of G/ML-CrI_3/Gr.** (a) STM image of G/ML-CrI_3/Gr ($V_s = 0.1$ V, $I_t = 0.8$ nA). The inset: a zoom-in STM image of the selected area (marked by dash square, $V_s = 1$ V, $I_t = 0.1$ nA) shows the CrI_3 lattice. The bottom graphite flake covered by graphene appears as the dark region on the left side of (a), while graphene covered monolayer CrI_3 appears as the bright region on the right side of (a). The line profile (red line) reveals a single-layer step with an apparent height of ~0.9 nm. (b) The dI/dV spectrum of G/ML-CrI_3/Gr (red solid line) taken in a large sample bias window ($-2.5\text{ V} \leq V_s \leq 2.1\text{ V}$), compared with the d$I$/d$V$ spectrum of G/FL-CrI_3/Gr (blue dashed line). (c) The dI/dV spectrum of G/ML-CrI_3/Gr (red solid line) taken in a small sample bias window ($-1.2\text{ V} \leq V_s \leq 0.42\text{ V}$), compared with the d$I$/d$V$ spectrum of G/FL-CrI_3/Gr (blue dashed line).

3. Calculated DOS of G/ML-CrI_3 with different Hubbard U

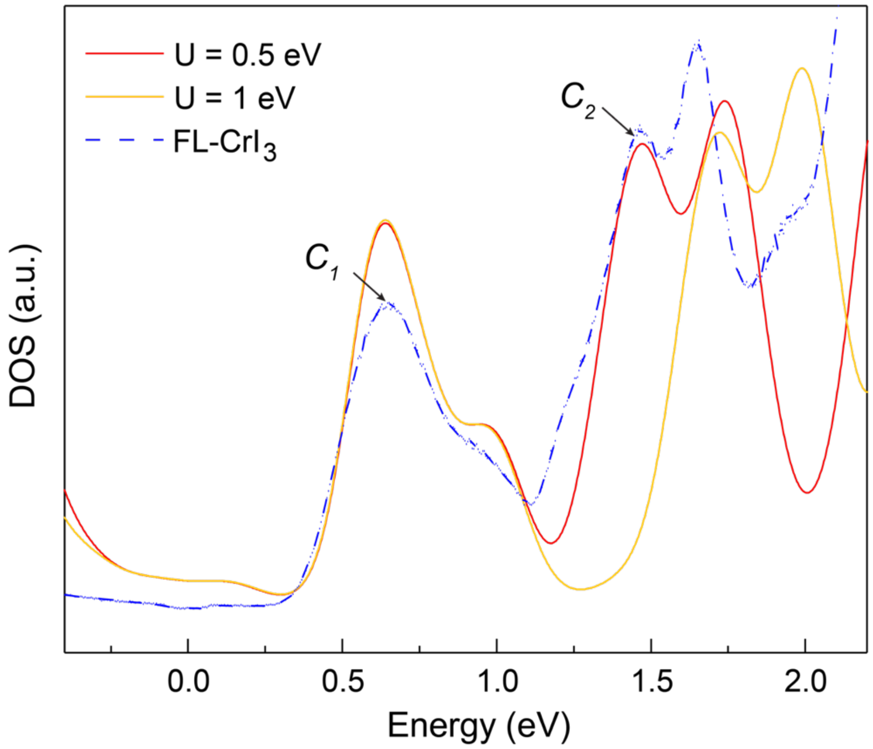

Figure S4. **Calculated DOS of G/ML-CrI_3 with a Hubbard U of 0.5 eV (solid red line) and 1 eV (solid yellow line) compared with the d*I*/d*V* spectrum of G/FL-CrI_3/Gr (dashed blue line).** Note, the calculated DOS of G/ML-CrI_3 is manually offset by 0.55 eV to align with the energy position of C_1 states resolved in d*I*/d*V* spectrum.

4. The transparency of graphene to tunneling electrons

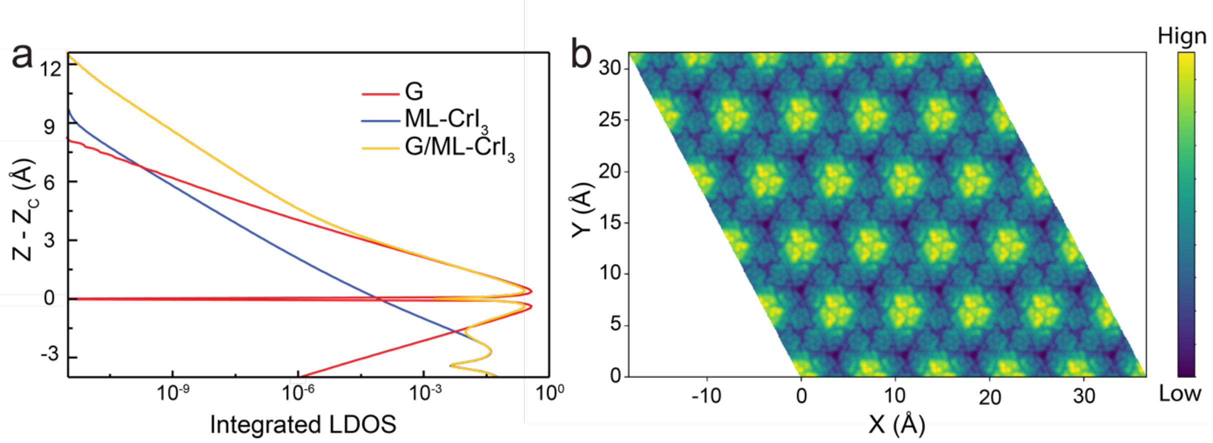

Figure S5. The transparency of graphene to tunneling electrons. (a) Integrated LDOS from E_F to $E - E_F = 3$ eV for the isolated graphene, ML-CrI3, and the hybrid G/ML-CrI3 as a function of vertical distance. All vertical distances are referenced to the graphene plane ($Z - Z_c = 0$ Å). (b) Simulated STM image of G/ML-CrI3 acquired at a vertical distance of $Z - Z_c = 6$ Å and $V_s = 3$ V.

With a few assumptions, the LDOS decays exponentially as a function of tip-sample distance z[1]:

$$LDOS\ (z) = C \cdot e^{-z/\lambda}$$

where C is a constant, λ is the decay length. The inverse decay length can be written as $\lambda^{-1} = 2\sqrt{\frac{2m\phi}{\hbar^2} + k_{||}^2}$, which is determined by the work function ϕ and the in-plane wave vector of the states $k_{||}$.

Here we have calculated the vertical decay of the integrated LDOS from E_F to $E - E_F = 3$ eV for the isolated graphene, ML-CrI3, and the hybrid G/ML-CrI3 heterostructure with a separation of 3.5 Å between graphene and CrI3 surface. The isolated ML-CrI3 is vertically offset to align with the CrI3 in the G/ML-CrI3 heterostructure. As shown in Figure S5a, the

graphene states undergo a much faster decay than ML-CrI_3 states with a crossover at 7 Å above graphene. It indicates graphene states have a small decay length which can be attributed to the large in-plane wave vector $k_{||}$ of graphene states[2]. Moreover, the vertical decay of integrated LDOS of the G/ML-CrI_3 hybrid system sets apart from that of graphene at the distance above 4 Å. When the vertical distance is larger than 4 Å ($Z - Z_c > 4$ Å), the effective decay length of the G/ML-CrI_3 system is nearly identical to the decay length of ML-CrI_3, which suggests that the integrated LDOS of G/ML-CrI_3 has a dominant contribution from the underlying ML-CrI_3. This is consistent with the feature (CrI_3 lattice) observed in the simulated STM image of G/ML-CrI_3 at $Z - Z_c = 6$ Å and $V_s = 3$ V (Figure S5b).

5. The moiré superlattice of G/FL-CrI_3/Gr at a twist angle φ = 16°

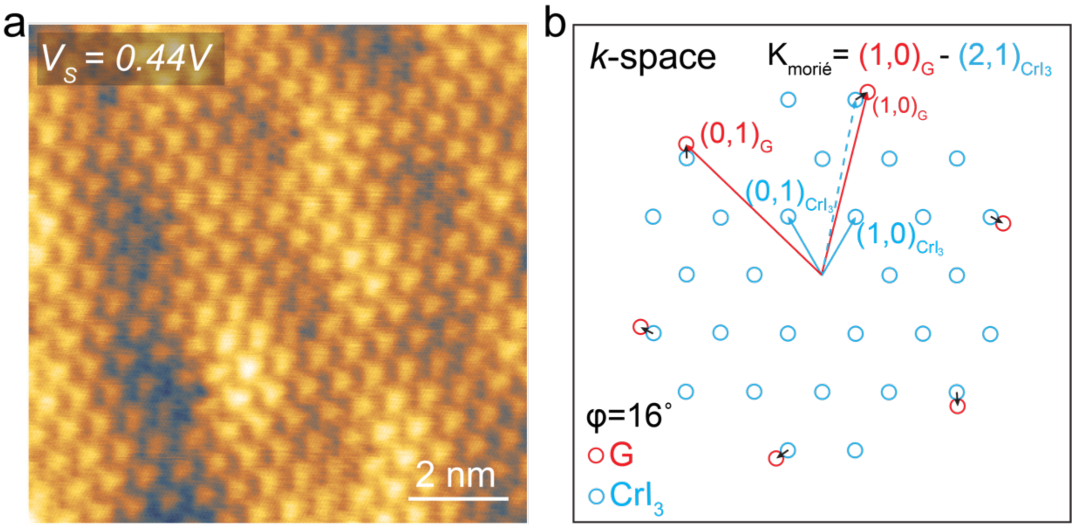

Figure S6. The moiré superlattice of G/FL-CrI_3/Gr at a twist angle φ = 16° between graphene and FL-CrI_3. (a) STM image (V_s = 0.44 V, I_t = 0.3 nA) in the same area of Figure 4a. (b) The reciprocal lattice of graphene (red circle) and CrI_3 (blue circle) at a twist angle φ = 16°.

We performed the geometrical analysis of the moiré superlattice in G/FL-CrI_3/Gr. A combination of Figure S6a and Figure 4a reveals a twist angle of $\varphi = 16°$ between the graphene and FL-CrI_3. We are able to extract the periodicity and orientation of the moiré superlattice of G/FL-CrI_3 based on this twist angle, in good agreement with the experimental results.

It is noted the that reciprocal lattice vector of moiré pattern ($\vec{K}_{morié}$) can be regarded as the difference between the reciprocal lattice vector of graphene and CrI_3[3]. Figure S6b shows the reciprocal lattice vectors of graphene and FL-CrI_3 indexed by vectors $(r,s)_G$ and $(m,n)_{CrI_3}$. As shown in Figure S6b, the $(1,0)_G$- and $(2,1)_{CrI_3}$- spot almost overlap in *k*-space. The difference of two vectors leads to the reciprocal lattice vector of a possible moiré pattern as symmetrically equivalent to: $\vec{K}_{morié} = (1,0)_G - (2,1)_{CrI_3}$. As a result, $\vec{K}_{morié}$ leads to a moiré superlattice in real space where the moiré length is $|M_1| = |M_2| = 3.18$ nm and the

rotation angle between graphene and moiré superlattice is $\theta = 41°$. Meanwhile, we experimentally determine the periodicity and orientation of moiré superlattice from STM image (Figure 4a). The moiré length is measured to be $|M_1|' = |M_2|' = 3.14 \pm 0.01$ nm. The angle between graphene and moiré superlattice is measured to be $\theta' = 41°$. The simulated results are in good agreement with our experimental observation.

6. The magnetic field dependent dI/dV maps of G/FL-CrI_3/Gr

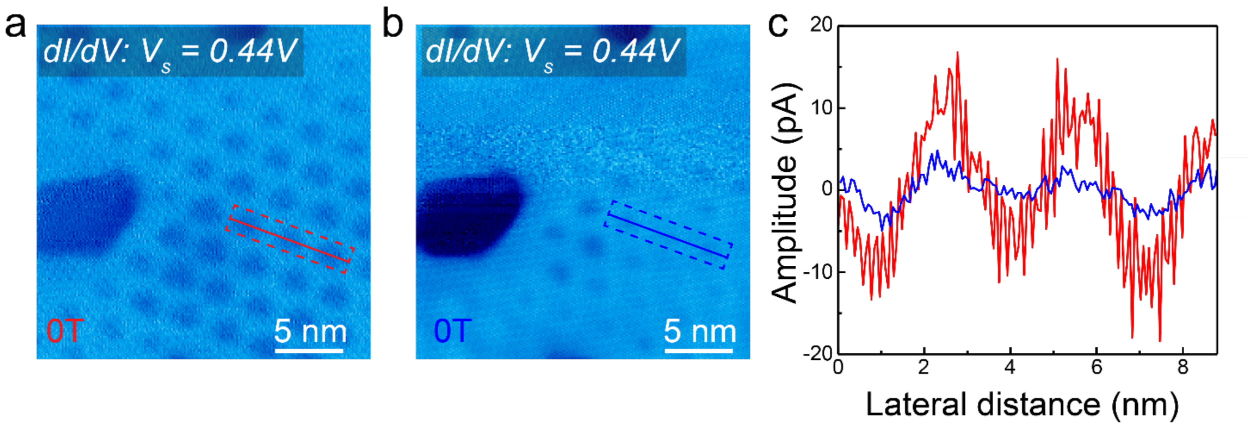

Figure S7. The amplitude of moiré contrast in the dI/dV maps of G/FL-CrI_3/Gr (V_s = 0.44 V, I_t = 0.5 nA). (a) The dI/dV map taken at the initial stage ($\mu_0 H = 0$ T) before ramping up the magnetic field. (b) The dI/dV map taken at the final stage ($\mu_0 H = 0$ T) after ramping down the magnetic field. (c) The line profiles across the indicated areas in the dI/dV maps. The difference between the maximum and minimum dI/dV signal is defined as the amplitude of moiré contrast, which is measured to be ~20 pA in image (a) and reduced to ~ 6 pA in image (b).

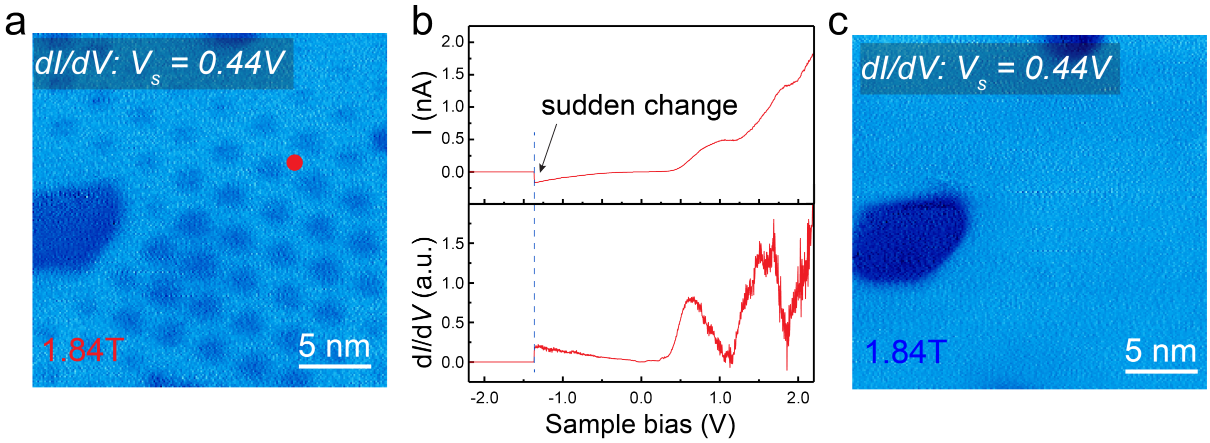

Figure S8. AFM-FM magnetic phase transition induced by the magnetic field with the assistance of tip-induced local gating. (a) The dI/dV map ($V_s = 0.44$ V, $I_t = 0.5$ nA) upon the application of 1.84 T magnetic field. We then took point dI/dV spectroscopy (the tip position is indicated by the red dot). (b) A sudden change of the I-V and dI/dV signal. (c) The dI/dV map ($V_s = 0.44$ V, $I_t = 0.5$ nA) taken after the sudden change of the dI/dV signal. The sudden change of I-V and dI/dV signal is likely to be associated with an abrupt AFM-FM magnetic phase transition induced by the magnetic field with the assistance of tip-induced local gating.

This can be attributed to the magneto-electric effect and the influence of doping on magnetization reported in previous studies[4-6].

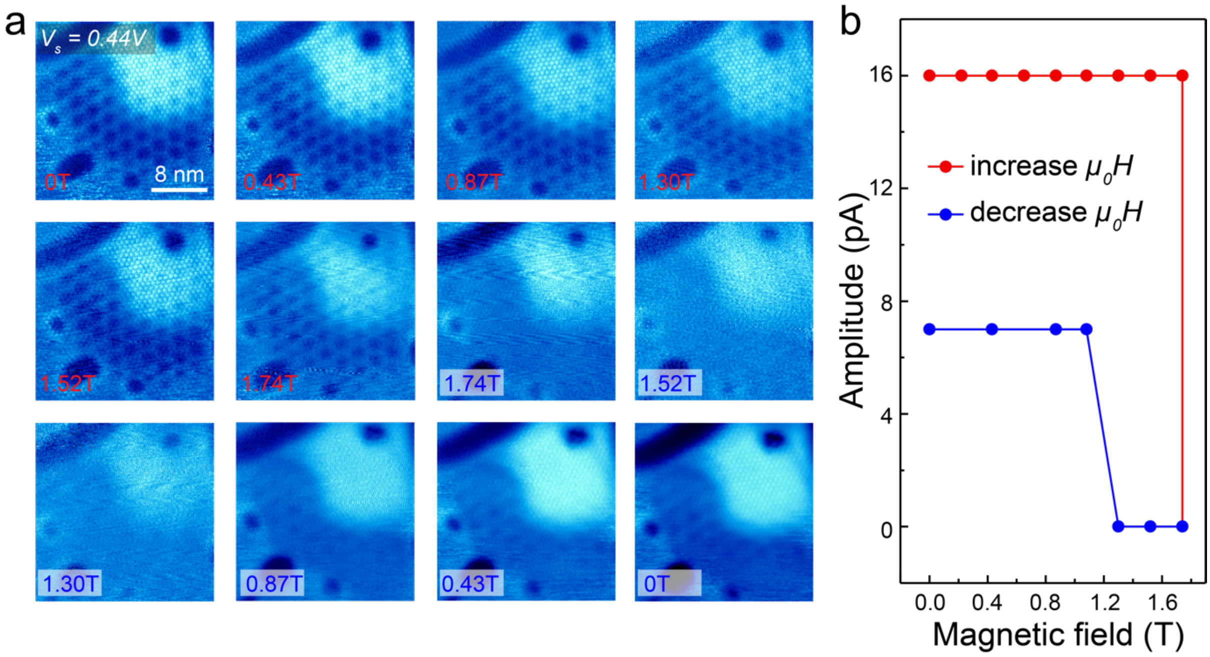

Figure S9. Magnetic field dependent moiré contrast in d*I*/d*V* maps. (a) Another set of magnetic field dependent d*I*/d*V* maps of G/FL-CrI_3/Gr (V_s = 0.44 V, I_t = 0.5 nA). The critical field was 1.74 T when moiré contrast disappeared. (b) Magnetic field dependent moiré contrast in the d*I*/d*V* maps taken at V_s = 0.44 V and I_t = 0.5 nA.

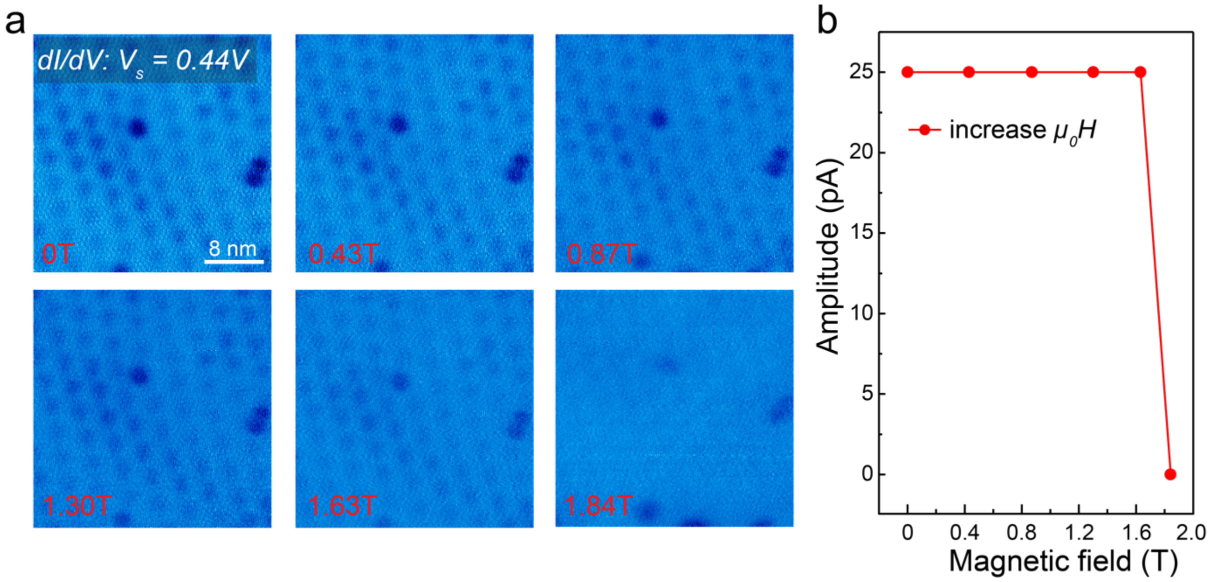

Figure S10. Moiré contrast in the d*I*/d*V* map vanished right after applying a magnetic field of 1.84 T in certain sample regions. (a) Magnetic field dependent d*I*/d*V* maps of G/FL-CrI_3/Gr (V_s = 0.44 V, I_t = 1 nA). (b) Magnetic field dependent moiré contrast in the d*I*/d*V* maps (V_s = 0.44 V, I_t = 1 nA).

7. The reported magnetic field for AFM-FM transition in FL-CrI_3

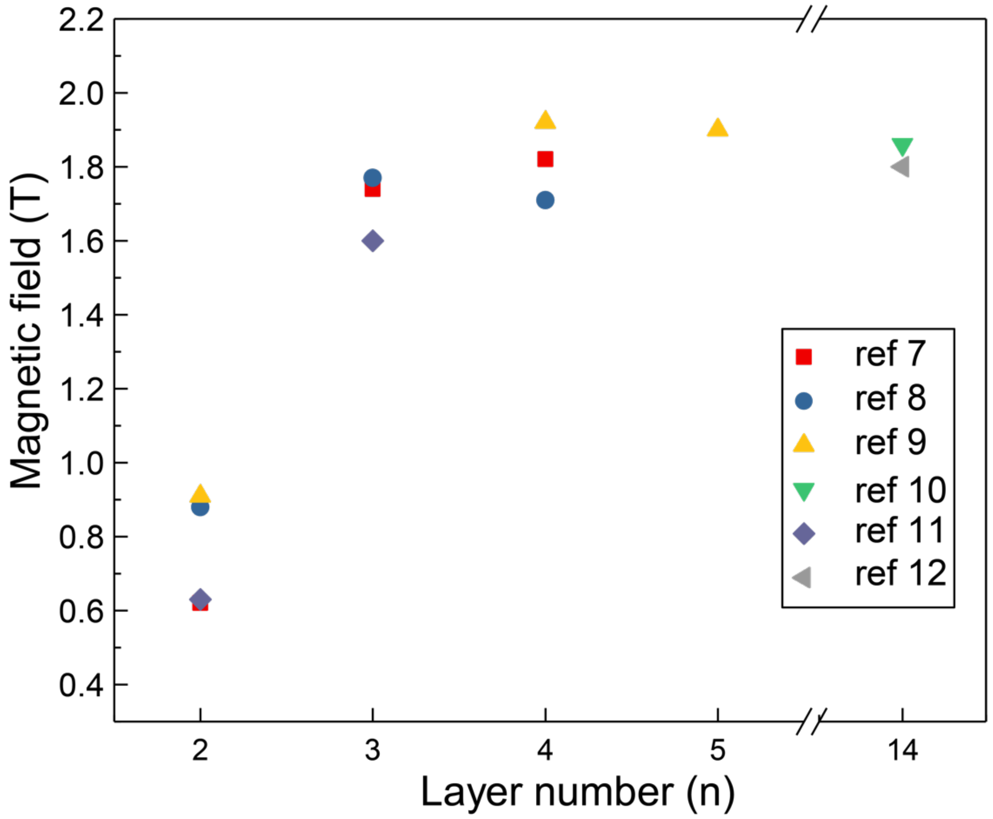

Figure S11. The typical magnetic field for AFM-FM transition on FL-CrI_3 in the previous reports[7-12]. The critical magnetic field for AFM-FM transition in thin CrI_3 flakes (layer number $n > 2$) is reported to be ranged from 1.60 T to 1.92 T (average value of 1.79 ± 0.10 T). The variation of the critical magnetic field is likely due to the sample-to-sample difference, the formation of domain structures and the influence of local environment[4,13,14]. Unfortunately, 1.84 T is the upper limit of the magnetic field in our system, which may be insufficient to flip the spin in certain sample regions. In such a case, AFM-FM phase transition could be triggered with the assistance of tip-induced local gating during dI/dV spectroscopic measurement, as illustrated in Figure S8. In addition, in the certain region of sample, we also observed that moiré contrast vanished in the dI/dV map immediately at a magnetic field of 1.84 T as shown in Figure S10.

8. The atomic registry dependent electronic structures of G/four-layer CrI_3

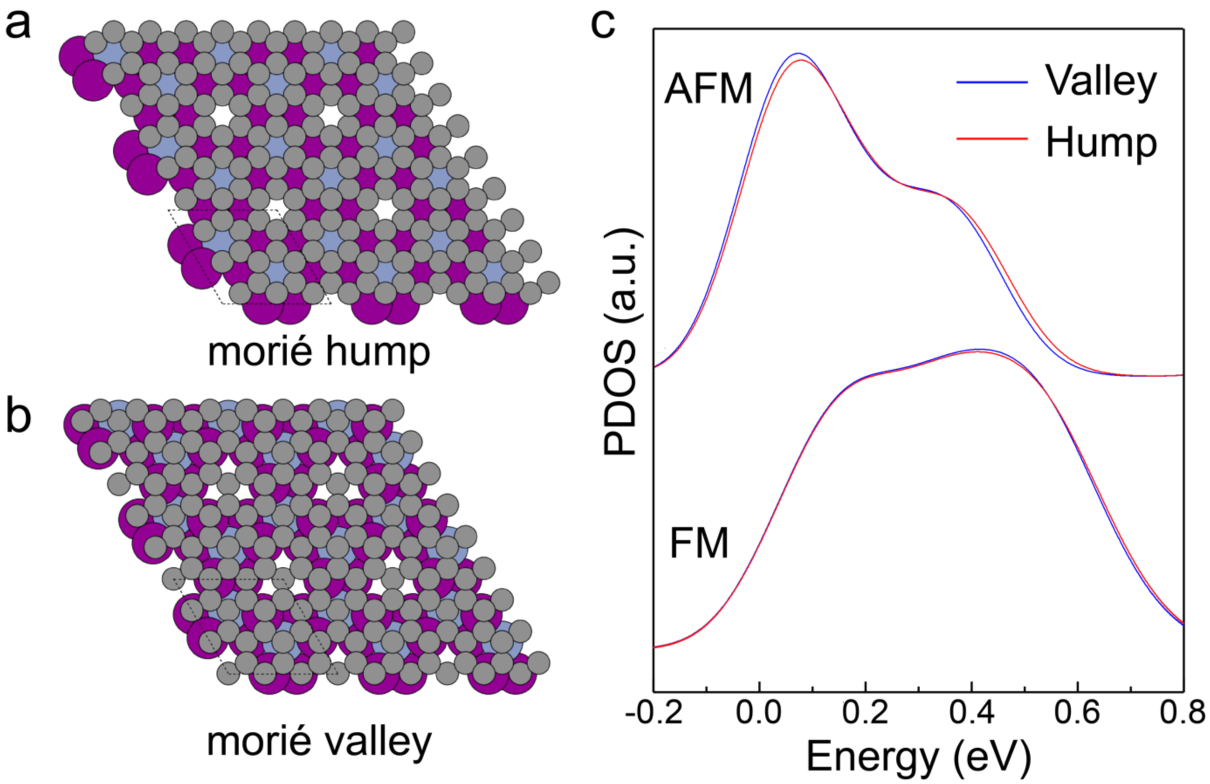

Figure S12. The atomic registry dependent electronic structures of G/four-layer CrI_3. (a) The atomic model of moiré hump. (b) The atomic model of moiré valley. (c) Calculated PDOS of top CrI_3 layer in moiré valley (blue) and moiré hump (red) when four-layer CrI_3 are AFM-coupled and FM-coupled.

A direct simulation of the large moiré superlattice of G/FL-CrI_3 at a twist angle $\varphi = 16°$ is computationally costly, beyond the capability of our computation power. To qualitatively demonstrate the atomic registry dependent electronic structure of G/FL-CrI_3 by DFT calculations, we use a (3×3) supercell of graphene placed on a single unit cell of four-layer CrI_3 with different atomic arrangements to mimic the local atomic registry in moiré hump and moiré valley separately (Figure S12a-b). Figure S12c compares the PDOS of top CrI_3 layer in moiré valley and moiré hump under different magnetic order. Specifically, when the four-layer CrI_3 is interlayer AFM-coupled, there is a small but noticeable difference between PDOS

in the moiré hump and valley. By contrast, PDOS of top CrI_3 layer are nearly same in moiré hump and valley when four-layer CrI_3 are interlayer FM-coupled.

9. Method for theoretical calculation

The DFT calculations were performed with the electronic structure software package GPAW[15,16] using the projector augmented wave method and a plane wave basis. The LDA+U functional was used in the Dudarev approach[17] with a value of U=0.5 eV for the Cr *d*-orbitals. The value of U was chosen in order to reproduce the distance between the C_1 and C_2 peaks obtained from STS. Unless otherwise specified, for all calculations we considered a single unit cell of CrI_3 with (3 × 3) unit cells of graphene adsorbed. The CrI_3 layers were stretched by 7% in order to obtain a lattice match with the LDA-optimized (3 × 3) graphene unit cell. In all calculations, we employed a 12 × 12 Monkhorst-Pack grid of k-points, a Fermi smearing of 1 meV and a planewave cutoff of 600 eV. The structures were relaxed until all forces were less than 0.01 eV/Å. We considered the two adsorption geometries shown in Figure S12a-b. The graphene sheet shows a slightly stronger binding to CrI_3 in the hump geometry compared to that in the valley geometry. The binding energy difference between the two geometries is determined to be approximately 1 meV per C atom. All band structures, wavefunctions and projected density of states were performed with a non-selfconsistent inclusion of spin-orbit coupling[18].

The simulated STS and STM images were carried out with a modified Tersoff-Hamann approach[1] with the local density of stated (LDOS) being obtained as

$$LDOS(\boldsymbol{r}) = \sum_{nk} f_{nk} |\psi_{nk}(x, y, x)|^2 \delta(\varepsilon_{nk} - eV_{bias})\, e^{-\alpha w_{nk}},$$

where ε_{nk} is eigenenergy of the state ψ_{nk}, f_{nk} are the occupation numbers and

$$w_{n\boldsymbol{k}} = \sum_{ia} \left|\langle \psi_{n\boldsymbol{k}} | \varphi_i^{C,a} \rangle\right|^2$$

is the total weight of the state $\psi_{n\boldsymbol{k}}$ on the graphene sheet (the state $\varphi_i^{C,a}$ is atomic orbital *i* on carbon atom a). Thus the graphene states can be projected out of the LDOS by choosing a finite value of α.

Reference:

1 Tersoff, J. & Hamann, D. R. Theory and application for the scanning tunneling microscope. *Phys. Rev. Lett.* **50**, 1998 (1983).

2 González-Herrero, H. c. et al. Graphene tunable transparency to tunneling electrons: a direct tool to measure the local coupling. *ACS Nano* **10**, 5131-5144 (2016).

3 Zeller, P. & Günther, S. What are the possible moiré patterns of graphene on hexagonally packed surfaces? Universal solution for hexagonal coincidence lattices, derived by a geometric construction. *New J. Phys.* 16, 083028 (2014).

4 Jiang, S., Shan, J. & Mak, K. F. Electric-field switching of two-dimensional van der Waals magnets. *Nat. Mater.* **17**, 406-410 (2018).

5 Jiang, S., Li, L., Wang, Z., Mak, K. F. & Shan, J. Controlling magnetism in 2D CrI_3 by electrostatic doping. *Nat. Nanotech.* **13**, 549-553 (2018).

6 Jiang, S., Li, L., Wang, Z., Shan, J. & Mak, K. F. Spin tunnel field-effect transistors based on two-dimensional van der Waals heterostructures. *Nat. Electron.* **2**, 159-163 (2019).

7 Song, T. et al. Giant tunneling magnetoresistance in spin-filter van der Waals heterostructures. *Science* **360**, 1214-1218 (2018).

8 Klein, D. R. et al. Probing magnetism in 2D van der Waals crystalline insulators via electron tunneling. *Science* **360**, 1218-1222 (2018).

9 Li, T. et al. Pressure-controlled interlayer magnetism in atomically thin CrI_3. *Nat. Mater.* **18**, 1303-1308 (2019).

10 Zhong, D. et al. Van der Waals engineering of ferromagnetic semiconductor heterostructures for spin and valleytronics. *Sci. Adv.* **3**, e1603113 (2017).

11 Song, T. et al. Switching 2D magnetic states via pressure tuning of layer stacking. *Nat. Mater.* **18**, 1-5 (2019).

12 Wang, Z. et al. Very large tunneling magnetoresistance in layered magnetic semiconductor CrI_3. *Nat. Commun.* **9**, 1-8 (2018).

13 Huang, B. et al. Layer-dependent ferromagnetism in a van der Waals crystal down to the monolayer limit. *Nature* **546**, 270-273 (2017).

14 Thiel, L. et al. Probing magnetism in 2D materials at the nanoscale with single-spin microscopy. *Science* **364**, 973-976 (2019).

15 Enkovaara, J. e. et al. Electronic structure calculations with GPAW: a real-space implementation of the projector augmented-wave method. *J. Phys. Condens. Matter.* **22**, 253202 (2010).

16 Larsen, A. H. et al. The atomic simulation environment—a Python library for working with atoms. *J. Phys. Condens. Matter.* **29**, 273002 (2017).

17 Mosey, N. J. & Carter, E. A. Ab initio evaluation of Coulomb and exchange parameters for DFT+ U calculations. *Phys. Rev. B* **76**, 155123 (2007).

18 Olsen, T. Designing in-plane heterostructures of quantum spin Hall insulators from first principles: 1T′− MoS_2 with adsorbates. *Phys. Rev. B* **94**, 235106 (2016).
